# White Mustard (*Sinapis alba* L.) Oil in Biodiesel Production: A Review

**DOI:** 10.3389/fpls.2020.00299

**Published:** 2020-04-02

**Authors:** Petar M. Mitrović, Olivera S. Stamenković, Ivana Banković-Ilić, Ivica G. Djalović, Zvonko B. Nježić, Muhammad Farooq, Kadambot H. M. Siddique, Vlada B. Veljković

**Affiliations:** ^1^Institute of Field and Vegetable Crops, Novi Sad, Serbia; ^2^Faculty of Technology, University of Niš, Leskovac, Serbia; ^3^Institute of Food Technology, University of Novi Sad, Novi Sad, Serbia; ^4^Department of Crop Sciences, College of Agricultural and Marine Sciences, Sultan Qaboos University, Al-Khoud, Oman; ^5^Department of Agronomy, University of Agriculture, Faisalabad, Faisalabad, Pakistan; ^6^UWA School of Agriculture and Environment, The UWA Institute of Agriculture, The University of Western Australia, Perth, WA, Australia; ^7^Serbian Academy of Sciences and Arts, Belgrade, Serbia

**Keywords:** biodiesel, white mustard seed, oil recovery, transesterification, *Sinapis alba* L

## Abstract

White mustard (*Sinapis alba* L.) seed oil is used for cooking, food preservation, body and hair revitalization, biodiesel production, and as a diesel fuel additive and alternative biofuel. This review focuses on biodiesel production from white mustard seed oil as a feedstock. The review starts by outlining the botany and cultivation of white mustard plants, seed harvest, drying and storage, and seed oil composition and properties. This is followed by white mustard seed pretreatments (shelling, preheating, and grinding) and processing techniques for oil recovery (pressing, solvent extraction, and steam distillation) from whole seeds, ground seed or kernels, and press cake. Novel technologies, such as aqueous, enzyme-assisted aqueous, supercritical CO_2_, and ultrasound-assisted solvent extraction, are also discussed. The main part of the review considers biodiesel production from white mustard seed oil, including fuel properties and performance. The economic, environmental, social, and human health risk/toxicological impacts of white mustard-based biodiesel production and use are also discussed.

## Introduction

*Sinapis alba* L. (white or yellow mustard, also known as *Brassica hirta*) is an annual plant of the family Brassicaceae that originates from the Mediterranean region (Katepa-Mupondwa et al., 2005). It is found worldwide as a cultivated plant species as well as a weed. It is a winter–spring plant that can be grown in short cycles, commonly in rotation with other cereal crops, with the possibility of second-crop cultures (Falasca and Ulberich, 2011). In Europe, white mustard is the most used mustard species (Monsalve et al., 1993) and in North America, it is the only species in commercial production for the food processing and condiment industries (Katepa-Mupondwa et al., 2005).

White mustard has many cropping applications, including edible oilseeds (Raney et al., 1995), fast-growing salads (Rahman et al., 2018), condiments, fodder, and green manure (Krstić et al., 2010). The plant can extract toxic heavy metals from soil (Kos et al., 2003). Young seedling leaves, which are rich in vitamin A, C, and E, are edible as fresh and tasty salad leaves and have a medicinal value to purify blood (Rahman et al., 2018). White mustard seed has significant agronomic value due to its high protein and oil contents and low starch content (Balke and Diosady, 2000). Its well-balanced amino acid profile makes the seed an attractive source of food-grade proteins. It is widely used as a binding agent and protein extender in meat processing and for hot dog mustard, mayonnaise, and salad dressings. The seeds have strong disinfectant properties and can be used as a food preservative (Rahman et al., 2018). Its essential oil can be used to preserve foods due to its potent antimicrobial activity (Peng et al., 2014). Industrially, white mustard seed oil is used as a lubricant and for lighting (Falasca and Ulberich, 2011). Moreover, the seed is used in traditional medicine for its anti-tumor, antiviral, and analgesic activities (Peng et al., 2013); it also has expectorant, stimulant, and antimicrobial activities that are useful for digestive and respiratory ailments (Sujatha et al., 2013).

Recently, white mustard seed oil has garnered interest for its use as a feedstock for biodiesel production (Ahmad et al., 2008; Issariyakul et al., 2011; Issariyakul and Dalai, 2012; Ciubota-Rosie et al., 2013; Sáez-Bastante et al., 2016). The oil itself can be used as an alternative fuel (Nieschlag and Wolff, 1971; Alam and Rahman, 2013). In addition, oil meal—a byproduct of the biodiesel industry from white mustard seeds—can be used for animal feed (Thacker and Petri, 2009) or further extracted to produce additional oil, thus improving economic benefits.

This review focuses on biodiesel production from white mustard seed oil as a feedstock and starts by discussing the botany, cultivation, and use of white mustard plants, seed harvest, drying and storage, and seed oil composition, properties, and uses. This is followed by the pretreatment of white mustard seeds (shelling, preheating, grinding) and processing techniques for oil recovery (pressing, solvent extraction, steam distillation) from whole seeds, ground seeds or kernels, and oil meals (press cakes). Novel technologies, such as aqueous, enzyme-assisted aqueous, supercritical CO_2_, and ultrasound-assisted solvent extraction, are also covered. The main part of the review considers biodiesel production from white mustard seed oil, including its fuel properties and performance. The economic, environmental, social, and human health risk/toxicological impacts of white mustard-based biodiesel production and use are also discussed.

## White Mustard

### Mustard Production

**FIGURE 1:**
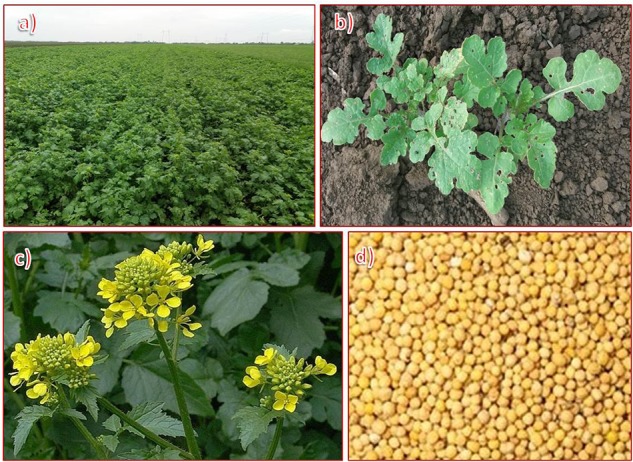
White mustard (*Sinapis alba* L.): **(a)** experimental field, **(b)** leaves, **(c)** flowers, and **(d)** seeds.

White mustard is an annual, broad-leaved, yellow-flowered, cool-season plant from the Brassicaceae family, that grows up to 100 cm high, with a relatively short growing season of about 85–95 days (Figure 1). The flowers, which bloom from May to June, are yellow with four petals. Mustard tolerates drought and heat, so it is well suited to production in drier soil zones. It is typically grown in rotation with cereal crops for its young leaves, seeds, or green manure. The use of white mustard in crop rotations is desirable due to its effect on residue conditions in the field, soil moisture conditions, and disease, weed, and insect problems. Ideally, white mustard is grown after a cereal crop. Mustard is commonly grown on summer fallow or stubble in dry and moist areas, respectively. Varietal selection involves various factors, including expected price, yield potential, and agronomic characteristics.

Young mustard leaves have a sharp flavor and are used in salads. The seeds are yellow to yellowish-brown and reveal an odor only when mixed with liquid. The heat and aroma of mustard come from sinalbine (a glycoside) and the essential oil, respectively. The advantages of cultivating white mustard as green manure include the long-term supply of soil organic matter, stable soil structure, and increased soil fertility, capacity for soil water storage, humus content, and soil microorganism activity.

### White Mustard Seed Production

**FIGURE 2:**
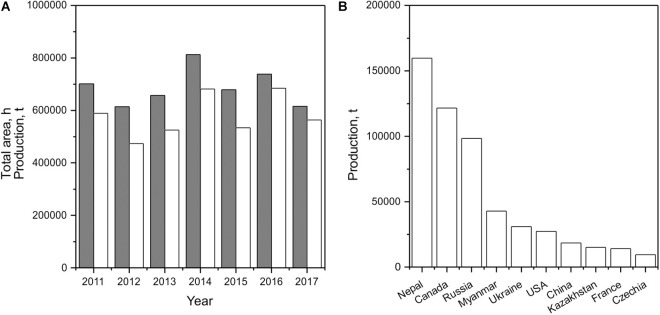
Mustard seed production in the world **(A)** and the world’s top producing countries **(B)** (gray, total area in ha; white, production in tons; source: http://www.fao.org/faostat/en/).

White mustard is globally cultivated on 60,000–80,000 ha annually, producing up to 685,000 t of seed (Figure 2A). Nepal and Canada are the world’s top mustard seed producers, with 159,710 t and 121,600 t, respectively, in 2017 (Figure 2B); these numbers represent around 27.6% and 21.0%, respectively, of world supply. Therefore, the global average production yield of white mustard seeds is 770–930 kg/ha. In Serbia, the average yield of mustard seeds is 1,500–1,800 kg/ha, with yields often exceeding 2,000 kg/ha. In comparison, the average yields of corn and rapeseed from 2015–2018 were 4,500 kg/ha and 2,800 kg/ha, respectively.

The amount of nitrogen fertilizer applied to the soil has a greater effect on grain yield and harvest index in white mustard than plant density (Sáez-Bastante et al., 2016). White mustard produces less oil per hectare than rapeseed or soybean. In addition, nitrogen fertilizer dose has been positively correlated with total oil extracted. Both plant density and nitrogen fertilizer dose influence fatty acid composition.

Knowledge of thermal and physical properties is essential for identifying appropriate processing equipment and optimizing transport and storage conditions. Additionally, specific heat capacity, thermal conductivity, and thermal diffusivity are important for determining the sensory quality of food products (Ikegwu and Ezeh, 2012; Mahapatra et al., 2013) and heat transfer characteristics (Sirisomboon and Posom, 2012; Jibril et al., 2016). Furthermore, the physical properties (bulk density, true density, porosity, surface area, length, and width) of food products and mechanical behavior under compression are needed to design processing equipment and identify the optimal conditions for harvesting, handling, sorting, storing, and processing (heating, drying, and cooling) seeds (Tavakoli H. et al., 2009; Tavakoli M. et al., 2009; Sangamithra et al., 2016). The thermal properties of white mustard seeds are cultivar-dependent, but the mechanical properties are not (Ropelewska et al., 2018). The minimum force required to break the white mustard seed coat and the average deformation are 17.5 N and 0.21 mm, respectively (Szczyglak and Zuk, 2012). Excessive breaking or deforming force reduces the quality of the processed seeds and increases shelling costs.

## White Mustard Oil Production

**FIGURE 3:**
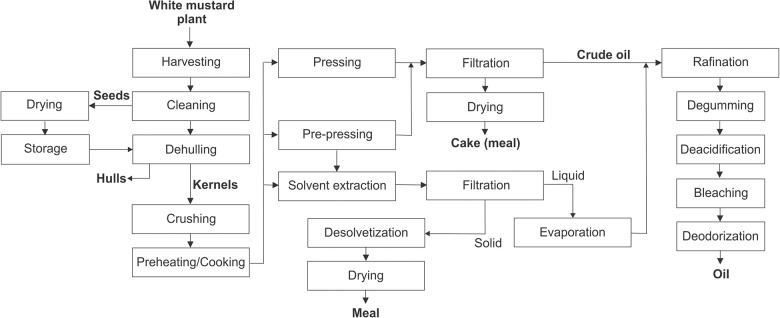
Schematic presentation of white mustard seed processing.

The overall process of white mustard oil production consists of seed harvesting, pre-cleaning, drying, storage and pretreatment, and oil recovery, refinement, and packaging. White mustard seed processing is schematically presented in Figure 3.

### Harvesting, Drying, and Storage of White Mustard Seed

White mustard plants should be harvested when the leaves start to yellow, and the pods start to turn brown (McKenzi and Carcamo, 2010). The pods must not be left on the plant for too long as they shatter when fully ripe, resulting in the loss of seed. Since white mustard plants are relatively resistant to pod shattering, they can be swathed or straight combined (McKenzi and Carcamo, 2010). For swathing, at least 75% of the seeds should be yellow (Sask Mustard, 2019). Harvesting can be undertaken manually using sickles or with a combine harvester. The seeds are either removed from the pods by hand, or the flower heads are placed in paper bags for a couple of weeks prior to seed maturity. A gentle shake of the bags releases most of the seeds. Modern combines have eliminated the need for hand-cutting plants. The use of a swath roller minimizes seed losses from wind damage by compacting the swath. When threshing with a combine, the lowest cylinder speed should be used to reduce pod cracking. Threshing should be carried out when the seed moisture content is less than 9.5% (McKenzi and Carcamo, 2010).

After seed harvest, caution is needed to preserve the oil quality by avoiding fat breakdown. Seeds are first pre-cleaned from external impurities, such as dust, plant leaves, stones, and ferrous particles (ABC Machinery, 2019). The removal of impurities allows for a high-quality product and prolonged life. For safe storage, seeds are commonly dried to remove water and to ensure high-quality oil recovery. Seeds are either dried by the sun or using hot-air convective drying—air and seed temperatures should not exceed 65°C and 45°C, respectively (Sask Mustard, 2019). Seeds are dried to less than 9% moisture content (McKenzi and Carcamo, 2010).

Dried seeds can be stored below 18°C for an extended period with appropriate aeration and precautions against rodent and insect infestation (Sask Mustard, 2019). Adequate ventilation or aeration of seeds during storage will maintain a low moisture content and reduce the risk of microbial development and general seed deterioration. Some companies crush and grind the seeds with roller mills before solvent extraction, which is then passed through sieves to separate the shells and bran.

### Pretreatment of White Mustard Seeds

In traditional processing, white mustard seeds are crushed and ground to a flour. In industrial oil production, the pretreatment involves shelling (decortication) and preheating (ABC Machinery, 2019). The oil-bearing portion of the seed (kernels) is separated from the shell by hand or a shelling machine, which gently cracks the seeds. The kernels are preheated by roasting or cooking to liquefy the oil in the oily organs and facilitate its release during recovery. Preheating increases the oil yield and protein availability in the seed cake. Machines offer easier and faster production of white mustard oil than manual operations. Before oil extraction, the kernels are usually ground to increase surface area and maximize oil yield.

### Oil Recovery From White Mustard Seed

White mustard oil is recovered from the seed either by pressing (expelling), solvent extraction preceding grinding, enzymatic extraction, aqueous extraction, or a combination of pressing and solvent extraction (prepress–solvent extraction), while steam distillation is rarely used. An overview of the methods used for the recovery of the white mustard oil is in Table 1. While whole seeds are used for mechanical oil extraction, white mustard seeds are usually dried and ground before solvent extraction or steam distillation. After recovery, the solvent is commonly removed from the oil by vacuum evaporation. The pressed oil is then filtered and dried by heating or vacuum evaporation. Sometimes, the white mustard seed oil is subjected to acid degumming, neutralization, and solid separation. The white mustard oil machine has made oil extraction easier, thus making the oil more affordable.

**TABLE 1:** Summary of white mustard seed oil extraction methods and results.

**Method**	**Material**	**Pretreatment of seed**		**Extraction**	**Refinement**		**References**
		**Drying**	**Milling**	**Technique/solvent/yield/time**	**Solvent or solid residue removal**	**Drying**	
Pressing	Seed	No	No	Press with 8 mm nozzles/13.28%	Vacuum filtration	Vacuum evaporation	Stamenković et al., 2018
	Seed	Oven (<70°C)	Grinder cutting (0.13–0.50 mm)	Pressing (5–8 kg/h)/oil content of cake reduced to 8–9%	Acid degumming and chemical deacidification, followed by centrifugation	Vacuum evaporation	Ciubota-Rosie et al., 2013
	Seed	–	No	Press extractor	Filtration	–	Mejia-Garibay et al., 2015
	Seed	–	No	Electric oil expeller	Electric filter apparatus	Heating at 120°C using a hot plate	Ahmad et al., 2013
	Seed	–	No	Electric oil expeller	Suction filtration	Heating above 100°C for 1 h	Sultana et al., 2014
	Seed	–	No	Pressing while applying heat (drive speed setting: 5–6)	Fiberglass filter disk under vacuum	–	Nie et al., 2016
Solvent extraction, batch	Seed (moisture contents: 3.78%)	No	Electric milling (1 min) before extraction (mean particle diameter: 0.44 mm)	Soxhlet apparatus/*n*-hexane (seed:solvent 1:10 g/mL)/20.64%/6 h	Vacuum filtration	Vacuum evaporation	Stamenković et al., 2018
			Manual crushing and electric milling (1 min) of press cake (mean particle diameter: 0.47 mm)	Soxhlet apparatus/*n*-hexane (seed:solvent 1:10 g/mL)/8.58%/6 h			
	Seed	Oven (<70°C)	Grinder cutting (0.13–0.50 mm)	Soxhlet apparatus/*n*-hexane (55°C)/39.2%/6 h or 43.5/18 h	Acid degumming and chemical deacidification, followed by centrifugation	Vacuum evaporation	Ciubota-Rosie et al., 2013
	Seed	–	Pestle and mortar	Soxhlet apparatus/*n*-hexane/31.6%/24 h	–	Rotary evaporation under vacuum	Singh et al., 2014
	Seed	–	Coffee mill	Soxhlet apparatus/*n*-hexane (70°C; 10:1 v/w)/25.30%/8 h	Anhydrous magnesium sulfate placed over a filter paper	Rotary evaporator at 40°C	Kozlowska et al., 2016
				Extraction (Folch method, room temperature, shaking)/ chloroform/methanol (2:1 v/v; 10:1 v/w)/29.80%/2 h	Whatman No. 1 paper filter into a separatory funnel with 1 M KCl solution; after gentle shaking, mixture left overnight for separation into two phases	Rotary evaporation under vacuum at 40°C	
	Seed	–	Pestle and mortar	Soxhlet apparatus/*n*-hexane	–	Rotary evaporation	Sáez-Bastante et al., 2016
	Dry, clean seeds	–	Crushing	Soxhlet apparatus/petroleum ether (60–80°C)/32.5%	–	Vacuum evaporation (<40°C)	Ali and McKay, 1982
	Seed	Dried overnight (50°C)	–	Soxhlet apparatus/petroleum ether (60°C, seed:solvent 5 g:100 mL)/21.1 ± 0.4%/16 h	–	Evaporation	Yaniv et al., 1994
	Seed	–	Coffee mill	Smalley-Butt apparatus/*n*-hexane/∼30%/4 h+2 h	–	Vacuum evaporation	Seal et al., 2008
	Seed	–	Grinder	Magnetically stirred beaker/*n*-hexane/overnight	Vacuum filtration	Vacuum evaporation	Nie et al., 2016
Solvent extraction, continuous	Seed	–	Grinding (0.2–0.3 mm)	Continuous stirred extractor (10:1 *n*-hexane:seed mass, 29°C, 30 h)/78% oil recovery	–	–	Ciubota-Rosie et al., 2009
Aqueous extraction	Dehulled flour	–	–	4:1 water:flour (g/g), 3 min blending, pH 11.00 ± 0.05, room temperature, 30 min extraction, centrifugation (∼9000 *g* for 20 min), re-extraction/washing stage of solid residue under same conditions/75% oil recovery	–	–	Balke, 2006
	Partially dehulled flour	–	–	Procedure of Balke (2006) (39% oil recovery in emulsion) and oil extraction from emulsion using isopropyl alcohol: Single extraction (25:1–31:1 isopropyl alcohol:oil mass)/90–94% oil recovery	–	–	Ataya Pulido, 2010
				Three-stage extraction (3:1 water:flour)/94% oil recovery			
				Four-stage extraction (2:1 water:flour)/96.3% oil recovery			
	Pre-ground dehulled flour (<100 mesh)	–	–	Procedure of Balke (2006) Four-stage PSE using fresh isopropyl alcohol at each stage (2:1 solvent:oil)/92.3% oil recovery			Jung and Diosady, 2012
				Four-stage PSER reusing extracted water-rich phase, containing isopropyl alcohol (2:1 solvent:oil)/84.0% oil recovery			
	Dehulled flour	–	–	Procedure of Balke (2006) Fully alkaline two-stage aqueous extraction (64.6% oil recovery in emulsion) and oil extraction from emulsion using cyclic ethers.	–	–	Tabtabaei and Diosady, 2012
				Tetrahydrofuran (4:1)/97% oil recovery and 5% water			
				1,4-dioxane (9:1)/95% oil recovery and 99% water			
	Dehulled flour	–	–	Procedure of Balke (2006) (57.5% oil recovery in emulsion) and oil extraction from emulsion using organic solvents with partial solubilities for oil	–	–	Tabtabaei et al., 2013
				30:1 dimethylformamide:oil mass/38% oil recovery			
	Dehulled flour	–	–	Fully alkaline two-stage aqueous extraction (flour slurry, 4:1 water:flour mass) + three-stage emulsion extraction with:	–	–	Tabtabaei et al., 2014
				0.75:1 tetrahydrofuran/100% oil recovery and 3.5% water			
				0.5:1 1-4-dioxane/85.8% oil recovery and 76% water			
	Dehulled flour (<100 mesh)	–	–	Fully alkaline two-stage aqueous extraction (flour slurry, 4:1 water:flour mass) + emulsion extraction with tetrahydrofuran (0.5:1 and 0.75:1, producing miscella I and II, respectively) (Tabtabaei et al., 2014) + adsorption (zeolite 4A)	–	–	Tabtabaei et al., 2015
				Single-stage aqueous extraction with tetrahydrofuran (4:1, producing miscella III) (Tabtabaei and Diosady, 2012) + adsorption (zeolite 4A)			
				Batch adsorption (10:1.5 miscella:zeolite, shaking rate 125 cycles/min), water removal/time: miscella I 72.4%/40 min, miscella II 98.8%/4 h, and miscella III 98.7%/4 h			
				Fixed-bed adsorption (2.5 cm column dimeter, 30 cm length), water removal/breakthrough time/bed capacity: miscella I (flow rate 1.6 mL/min) 100/44.6 h/0.222 g/g; miscella II (flow rate 2.0 mL/min) 98.6%/14.2 h/0.244 g/g			
Enzymatic aqueous extraction	Dehulled flour	–	–	Sequential two-stage aqueous extraction (flour slurry, 4:1 water:flour mass): (1) pH 4.8–5.0, 25°C, 30 min; (2) pH 11, 30 min, room temperature/30.0% oil, 58.7% water, 8.3% protein, and 5.2% phospholipids	–	–	Tabtabaei and Diosady, 2013
				Sequential two-stage enzymatic aqueous extraction: (1) pH 4.8, 40°C, 3% carbohydrase of flour mass (Viscozyme L, Pectinex Ultra SP-L, Celluclast each 1%), 3 h; (2) pH 11, room temperature, 3 h/35.3% oil, 52.2% water, 9.1% protein, and 5.9% phospholipids			
				Sequential two-stage aqueous extraction+emulsion extraction: (3) 3:1 water:emulsion mass, pH 11, 25°C, 30 min/80.0% oil, 20.6% water, 0.6% protein, and 0.6% phospholipids			
				Sequential two-stage aqueous extraction+emulsion extraction: (4) 3:1 water:emulsion mass, pH 11, 25°C, 3 h/80.1% oil, 20.8% water, 0.7% protein, and 0.8% phospholipids			
				Fully alkaline two-stage aqueous extraction (Tabtabaei and Diosady, 2012)/55.5% oil, 39.8% water, 3.2% protein, and 3.3% phospholipids			
Ultrasound-assisted extraction	Seed	Dry	Grinding (18 mesh)	Flask in ultrasonic batch (room temperature)/diethyl ether (10 g seed:40 mL solvent)/8.96%/30 min	Filtration under reduced pressure	Evaporation	Peng et al., 2013
Supercritical CO_2_ extraction	Seed	–	Mill equipped with 1.0-mm sieve	Single extraction (2 mL CO_2_/min, 51.7 MPa, 100°C, 60 min)/21.59 ± 0.29%	–	–	Barthet and Daun, 2002
				Multiple extraction (5 × 20 min)/28.63 ± 0.57%			
				Multiple extraction with modifier (2 × 30 min + 30 min with 15% ethanol)/28.60 ± 0.49%			
	Seed	–	Mill with diatomaceous earth	Single extraction (41.4 MPa, 80°C, 90 min)/∼30%	–	Vacuum evaporation	Seal et al., 2008
				Two-step extraction with modifier (60 min with 15% ethanol)/∼30%			
Pressing + solvent extraction	Seed	Oven (<70°C)	Grinder cutting (0.13–0.50 mm)	Pressing (5–8 kg/h) + solvent extraction (*n*–hexane)/>40% (92% of total oil)/2 h	Acid degumming and chemical deacidification, followed by centrifugation	Vacuum evaporation	Ciubota-Rosie et al., 2013
	Seed	Seed moisture content: 3.78%	Electric milling (1 min) of seeds (mean particle diameter: 0.44 mm)	Seed pressing + Soxhlet extraction (*n*-hexane; 1:10 g/mL seed:solvent)/19.90%/6 h	Vacuum filtration	Vacuum evaporation	Stamenković et al., 2018
			Manual crushing and electric milling (1 min) of press cake (mean particle diameter: 0.47 mm)	Seed pressing + maceration (*n*-hexane; 70°C; 1:6.5 g/mL seed:solvent)/20.48%/5 min			
				1:8.5 g/mL seed:solvent/20.50%/5 min			
Steam distillation	Seed	Dry	Grinding (18 mesh)	Steam distillation/water vapor/6.48%	–	–	Peng et al., 2013

#### Oil Pressing

Oil pressing extracts oil from whole seeds by physical (mechanical) pressing. Mechanical oil recovery from whole white mustard seeds involves cold pressing (Ciubota-Rosie et al., 2013; Stamenković et al., 2018), hot pressing (Nie et al., 2016), or expelling (Ahmad et al., 2013; Sultana et al., 2014). Oil pressing has many advantages, including simplicity, ease of operation, flexibility, and fewer processing operations and machines, and produces good quality oil and cake. However, it is less efficient than the Soxhlet extraction (Stamenković et al., 2018). The cake from oil pressing contains up to 8–9% oil (Ciubota-Rosie et al., 2013). A commercial white mustard oil pressing line usually includes a sheller, cleaning sieve, cooker, oil expeller, and filter (ABC Machinery, 2019).

#### Solvent Oil Extraction

For solvent extraction, white mustard seeds are dried, ground, and then subjected to extraction. Various solvents and extraction techniques are used, including the Soxhlet extraction with petroleum ether (Ali and McKay, 1982; Yaniv et al., 1994) or *n*-hexane (Seal et al., 2008; Ciubota-Rosie et al., 2013; Singh et al., 2014; Kozlowska et al., 2016; Sáez-Bastante et al., 2016; Stamenković et al., 2018), Smalley-Butt extraction (Seal et al., 2008), traditional maceration with *n*-hexane (Stamenković et al., 2018), shaking extraction using chloroform/methanol (Kozlowska et al., 2016), continuous one-step maceration with *n*-hexane (Ciubota-Rosie et al., 2009), ultrasonic extraction with diethyl ether, ethyl acetate, and petroleum ether (Peng et al., 2013), supercritical CO_2_ extraction (Barthet and Daun, 2002; Seal et al., 2008), aqueous extraction (Ataya Pulido, 2010; Jung and Diosady, 2012; Tabtabaei and Diosady, 2012, 2013; Tabtabaei et al., 2014), and enzymatic aqueous extraction (Tabtabaei and Diosady, 2013). After extraction, the solvent is usually removed from the oil by evaporation under reduced pressure. Besides the Soxhlet apparatus, the Butt tube extractor (Seal et al., 2008) and the FOSFA method (Barthet and Daun, 2002) have been used to measure oil content in white mustard seeds.

##### Soxhlet extraction

The Soxhlet extraction is a traditional method for oil extraction that has several disadvantages, including the use of costly, non-selective, hazardous, and flammable high-purity organic solvents, toxic emissions during extraction, and a laborious and time-consuming procedure (Gayas and Kaur, 2017). However, this method provides the highest oil yields from ground white mustard seeds due to the high solvent:seed ratio (usually 10:1 mL/g), long processing time (usually 6 h or longer), and high extraction temperature (boiling point). Using *n*-hexane (53°C), Ciubota-Rosie et al. (2013) reported the highest oil yields of 39.2% and 43.5% after 6 h and 18 h of Soxhlet extraction, respectively, while much lower oil yields (21–32%) have been reported by other researchers (Seal et al., 2008; Singh et al., 2014; Stamenković et al., 2018). Petroleum ether extractions resulted in oil yields of 21.2 ± 0.4% from the 11 best lines of white mustard (Yaniv et al., 1994). Soxhlet extraction from a press cake yielded 8.58% oil, which was 41.6% of the oil content from the processed seed (Stamenković et al., 2018). Differences in oil yields of white mustard seeds using Soxhlet extraction are attributed to variations in oil content in cultivars of different geographic origin, particle sizes of processed ground seeds, solubilities of solvents used, and extraction temperatures.

#### Aqueous Extraction

Aqueous extraction uses water as an extracting solvent to recover oil from oilseeds. It is emerging as an alternative to hexane extraction due to fewer related health, safety, and environmental problems (Rosenthal et al., 1996). It is also beneficial for the simultaneous recovery of oil and high-quality proteins for industrial applications (Tabtabaei and Diosady, 2012). However, low oil yields and the stable emulsion formation have prevented the commercialization of aqueous extraction because an additional demulsification step is needed to recover the oil fully.

Many researchers have used aqueous oil extraction from white mustard seed and flour. Using a two-step process, Balke (2006) achieved the highest oil and protein yields of 85% and 95%, respectively, from fully dehulled white mustard flour. Ataya Pulido (2010) recovered only 39% oil from partially dehulled flour in the form of an oil-in-water emulsion. The low oil yield was ascribed to mucilage, a polysaccharide present in the tested flour, with good emulsifying properties, which prevented oil separation from the solids. Balke and Diosady (2000) developed a rapid aqueous extraction process for mucilage removal from whole white mustard seeds prior to grinding and oil separation. The seed coat, including mucilage, can also be removed readily by mechanical dehulling.

Oil-in-water emulsions are successfully destabilized with a freeze-thaw treatment, while a heat treatment followed by centrifugation and pH adjustment to the isoelectric point of white mustard protein could not break the formed emulsions (Ataya Pulido, 2010). Organic solvents could be used to fully or partially dissolve both oil and water to recover free oil from the emulsion, such as isopropyl alcohol (Ataya Pulido, 2010; Jung and Diosady, 2012) and dimethylformamide with partial solubilities for oil (Tabtabaei et al., 2013), and tetrahydrofuran and 1,4-dioxane with complete solubilities for oil (Tabtabaei and Diosady, 2012).

The cost-effective technologies for recovering miscella with high oil and low water contents from the emulsion are desirable. Tabtabaei et al. (2014) developed multi-stage extractions of the emulsion using lower mass ratios of tetrahydrofuran- or 1,4-dioxane:oil to produce miscellas with low water content. Using 0.5:1 and 0.75:1 tetrahydrofuran:oil mass ratios, 97% of the oil was recovered in the oil-rich miscella that contained only 1% and 1.5% water, respectively. Having low-free fatty acid and phosphorus contents, the produced miscella might be suitable feedstock for biodiesel production by direct base-catalyzed transesterification. Tetrahydrofuran—added to the oil-in-water emulsions—produces miscellas containing about 1–2% water, thus preventing the direct conversion of the miscella to biodiesel. These miscellas are successfully dehydrated by adsorption on zeolite 4A at room temperature using batch or continuous fixed-bed systems to the water content lower than 0.3% specified for biodiesel feedstock (Tabtabaei et al., 2014).

Based on the weaknesses of the aqueous extraction process mentioned above, an improved emulsion destabilization process that concurrently extracts oil and water in separate phases with enhanced solvent usage efficiency is needed. The reported results have shown that organic solvents with complete solubility for oil (*n*-hexane, petroleum ether, diethyl ether, and ethyl acetate) are more efficient for oil recovery from white mustard emulsion than those having partial solubility, providing high oil recovery at a lower solvent:oil mass ratio.

#### Enzymatic Aqueous Extraction

The stable oil-in-water emulsions produced by aqueous extraction can be destabilized using different enzymes (alone or in combination) that hydrolyze certain emulsifiers (Jeevan Kumar et al., 2017). This process, known as enzymatic aqueous extraction, is hampered by the high cost of enzyme production and downstream processing, long incubation time, and additional demulsification step.

Tabtabaei and Diosady (2013) applied a three-step process to recover the oil from dehulled white mustard flour using enzymatic aqueous extraction. In the first step, the flour slurry (4:1 water:flour weight ratio) was extracted in the presence of 3% carbohydrases (Viscozyme L, Pectinex Ultra SP-L, Celluclast) at pH 4.8 and 40°C for 3 h. The second step involved alkaline extraction (pH 11) of the solid residue at room temperature for 30 min. The third step was the extraction of oil from the collected emulsion using a 3:1 water:emulsion mass ratio at pH 11 and 25°C for 30 min. A two-stage alkaline aqueous extraction at pH 11 and sequential two-stage aqueous extraction at pH 4.8 and 11 were performed without enzymes using the same procedure. This alkaline treatment produced unstable emulsions and increased oil dispersion into the skim (to about 26%). The protease Protex 6 L treatment (2.5%) recovered >91% oil in the emulsions while the phospholipase treatment had no effect on free oil or protein recovery by isoelectric precipitation. However, the enzymatic aqueous extraction of dehulled white mustard flour does not offer adequate improvements in protein recovery to justify the additional effort and cost.

#### Novel Solvent Extraction Methods

Novel solvent extraction methods, such as ultrasound-assisted and supercritical CO_2_ extraction, have been rarely used for white mustard seed oil recovery, despite having numerous advantages over traditional methods related to time and energy consumption, safety hazards, low-quality oil and meal, environmental risks, and toxicological consequences (Reverchon and De Marco, 2006; Koubaa et al., 2016). Ultrasonication fragments or disrupts the seed particles immersed in the extraction vessel, thus accelerating diffusion, enhancing overall mass transfer, and reducing processing time and temperature (Koubaa et al., 2016). Peng et al. (2013) reported the greater efficiency of ultrasound-assisted oil extraction from white mustard seeds by diethyl ether (8.96% oil yield) than by ethyl acetate (7.63%) or petroleum ether (7.54%). The known benefits of liquid CO_2_ are its non-toxic and non-explosive nature, availability, ease of removal, and preservation of oil quality. Seal et al. (2008) used neat CO_2_ and a mix of CO_2_ and 15% ethanol to extract oil from white mustard seed using a modified two-step process. Both extraction fluids yielded about 30% oil, but the ethanol mix reduced the processing time. Barthet and Daun (2002) improved the efficiency of oil recovery from ground white mustard seeds using multiple extractions. Five consecutive extractions (5 × 20 min) without a modifier or a combination of double extractions with neat CO_2_ (2 × 30 min) followed by a third extraction with 15% modifier (30 min) produced higher oil yields (about 28.6%) than a single extraction (21.59%). However, the supercritical CO_2_ method extracted 25% less oil than the standard FOSFA method, suggesting seed matrix effects on the oil extraction.

#### Other Oil Extraction Methods

In addition to the above-mentioned major methods, the oil can be also recovered from white mustard seed by steam distillation, a two-step process combining oil pressing and solvent extraction and continuous single-stage solvent extraction. Steam distillation yielded 6.48% oil from white mustard seed, which was less than that from ultrasound-assisted extraction (8.96%) (Peng et al., 2013). However, steam distillation has high equipment costs, is time-consuming, and controlling the temperature is difficult (Gayas and Kaur, 2017). In the two-step process, seeds are first pressed to remove most of the oil and then the residual oil is extracted from the press cake using a solvent. Stamenković et al. (2018) combined pressing with either Soxhlet extraction or maceration using *n*-hexane to extract total oil yields of 19.6% and 20.5%, respectively. Ciubota-Rosie et al. (2013) reported that pressing followed by solvent extraction using *n*-hexane (for 2 h) yielded >41% oil and produced a press cake with low oil content (<2%). The continuous single-stage extraction recovers 78% oil from ground white mustard seeds using *n*-hexane (Ciubota-Rosie et al., 2009). This extraction was modeled using the generalized reduced gradient method to determine the optimum conditions for maximum efficacy.

### Comparison of Various Oil Recovery Methods

Excluding comparisons with standard methods, the various oil recovery methods have rarely been compared. For instance, Peng et al. (2013) reported higher efficiency of oil extraction using ultrasound-assisted extraction with diethyl ether than with steam distillation. Stamenković et al. (2018) compared cold pressing, Soxhlet extraction, and combined pressing and solvent extraction. Oil yields obtained from a Soxhlet extraction using *n*-hexane (25.30 ± 1.24%) for 8 h was lower than that from the Folch method (29.80 ± 2.95%) using chloroform/methanol (2:1, v/v) with 2 h shaking at room temperature (Kozlowska et al., 2016). This result was attributed to the extraction of polar materials (phospholipids), apart from neutral triacylglycerols. As shown in Table 2, seed cold pressing followed by press cake maceration under optimal extraction conditions recovered >99% oil, which was close to the reference Soxhlet method and much higher than that of seed cold pressing alone (41.6%).

**TABLE 2:** Oil yields obtained from various oil sources by different extraction methods.^a^

**Extraction method**	**Source**	**Oil yield^b^ (g/100 g)**	**%**
Soxhlet	Seed	20.64 ± 0.18	100.0
Soxhlet	Press cake	8.58 ± 0.06	41.6
Cold pressing	Seed	13,28 ± 0.11	64.3
Cold pressing/Soxhlet	Seed/press cake	19.90 ± 0.04	96.4
Cold pressing/maceration (70°C, 6.5:1, 5 min)	Seed/press cake	20.48	99.2
Cold pressing/maceration (70°C, 8.5:1, 5 min)	Seed/press cake	20.50	99.3

*^*a*^Adapted from Stamenković et al. (2018); ^*b*^Mean value ± standard deviation.*

Table 3 summarizes the results from a study by the University of Toronto on the destabilization of emulsions from dehulled white mustard flour using several methods. At lower solvent:oil mass ratios, the single extraction using tetrahydrofuran or 1,4-dioxane recovered more oil than dimethylformamide or isopropyl alcohol. Similar results were obtained with multiple-stage extractions using much lower solvent:oil mass ratios with both types of solvents. The use of recycled isopropyl alcohol recovered less oil than the other solvents, but the water content in the oil-rich phase decreased substantially due to improved oil and water separation. The isopropyl alcohol usage efficacy, as represented by oil extracted per isopropyl alcohol used, increased by a factor of 2.4 when the recycled solvent was used, which would reduce processing costs (Tabtabaei and Diosady, 2013).

**TABLE 3:** Summary of emulsion destabilization results.

**Extraction process^a^**			**Solvent:oil mass ratio**	**Recovery by final oil extract (%)**		**Final oil extract composition (%)**			**References**
	**Demulsification^b^**	**Solvent^c^**		**Oil recovery**	**Water recovery**	**Oil**	**Water**	**Solvent**	
Two-stage alkaline	None	None	–	–	–	57.5 ± 4.0	38.4 ± 3.9		Tabtabaei and Diosady, 2012
	Single extraction	Tetrahydrofuran	4:1	97.2 ± 0.9	<2	23	5	72	
		1,4-dioxane	9:1	95 ± 3	99	9	7	84	
		Dimethylformamide	30:1	38 ± 3	–	1	3	96	Tabtabaei et al., 2013
		Isopropyl alcohol	31:1	94.0	6.1	2.8	2.1	95.0	Ataya Pulido, 2010
	Three-stage extraction	Tetrahydrofuran	0.75:1	100	3.5	55.9 ± 1.5	1.5 ± 0.1	42.6 ± 1.6	Tabtabaei et al., 2014
		1,4-dioxane	0.5:1	85.9	0	86.5 ± 0.5	0.2 ± 0.0	13.3 ± 0.5	
	Four-stage extraction	Isopropyl alcohol	2:1	97	100.0	10.0	7.5	82.5	Ataya Pulido, 2010
		Isopropyl alcohol	0.2:1	92.3 ± 2.3	4.6 ± 0.6	21.1 ± 1.4	0.6 ± 0.1	78.3 ± 1.4	Jung and Diosady, 2012
		Isopropyl alcohol (recycled)	0.2:1	84.0 ± 0.9	1.0 ± 0.1	92.9 ± 0.8	0.6 ± 0.0	6.5 ± 0.8	
	None	None	–	<5 (pH 2–11)^d^ 94.7 ± 4.2^e^ 4.0 ± 0.2^f^	–	55.5 ± 4.6	39.8 ± 3.9	–	Tabtabaei and Diosady, 2013
Sequential two-stage	None	None	–	30 (pH 4–6)^d^ 92.7 ± 2.6^e^ 22.5 ± 0.7^f^	–	30.0 ± 2.5	58.7 ± 2.2	–	
	Single extraction	Water	3:1	96.5 (pH 4.5)^d^ 97.8 ± 0.9^e^ 99.4 ± 0.6^f^	–	80.0 ± 3.4	20.6 ± 3.3	–	
Sequential two-stage enzymatic	None	None	–	51 (pH 3)^d^ 91.3 ± 2.4^e^ 41.2 ± 1.7^f^	–	35.3 ± 2.1	52.2 ± 1.0	–	
	Single extraction	Water	3:1	91.1 (pH 4.5)^d^ 94.6 ± 0.1^e^ 94.3 ± 0.1^f^	–	80.1 ± 2.2	20.8 ± 3.8	–	

*^*a*^Two-stage alkaline extraction: Water:flour ratio: 4:1 g/g; blending time: 3 min; pH 11.00 ± 0.05; room temperature; extraction time: 30 min; centrifugation (∼9000 *g* for 20 min); re-extraction/washing stage of solid residue under same conditions; Sequential acid/alkaline two-stage: Slurry (water:flour ratio: 4:1 g/g) first extracted at native pH (4.8–5.0) and 25°C for 30 min followed by second-stage alkaline extraction (pH 11) for another 30 min at room temperature; Sequential two-stage – enzymatic: Slurry (water:flour ratio: 4:1 g/g) first extracted at pH 4.8 and 40°C in presence of 3% carbohydrases of the flour mass for 3 h, followed by second-stage alkaline extraction (pH 11) at room temperature for 30 min. ^*b*^None – no emulsion destabilization; Single extraction: single-stage emulsion destabilization by adding a solvent; multiple extractions: multiple-stage emulsion destabilization by adding a solvent. ^*c*^None – no solvent was added. ^*d*^After pH adjustment. ^*e*^After protease treatment (Protex 6 L, 2.5%, pH 9.0, 60°C, 3 h). ^*f*^After phospholipase treatment (G-ZYME G999, 2.5%, pH 7.5, 40°C, 3 h).*

### Mechanisms, Optimization, Kinetics, and Thermodynamics of Ground Press Cake Maceration

Only Stamenković et al. (2018) have studied the mechanisms, optimization, kinetics, and thermodynamics of oil extraction from ground press cake, remaining after pressing whole white mustard seeds by maceration using *n*-hexane.

#### Mechanism of Ground Press Cake Maceration

Typically, for oily plant material, maceration of ground press cake at a constant temperature increases oil yields rapidly within the first minute before decelerating (up to about the 5th min) to reach a plateau (next 10 min) (Stamenković et al., 2018). Maceration reached practical saturation within 5 min. The speedy first-extraction step (oil washing) grinds and washes out the oil from the external surfaces of seed particles. In the second step (oil diffusion), the oil diffuses from the interior of the particles and dissolves in the solvent.

#### Optimization of Ground Press Cake Maceration

A response surface 3D plot was used to visualize the effects of the process factors and their interactions on the oil yield obtained within 5 min (Stamenković et al., 2018). Generally, increasing both the extraction temperature and solvent-to-seed cake ratio increased oil yield. The maximum oil yield was achieved at an extraction temperature close to 70°C and a solvent:seed cake ratio of between 6.5:1 and 8.5:1 mL/g. Taking the lowest solvent amount as the criterion of choice, the selected optimal extraction conditions were 6.5:1 mL/g, 70°C, and 5 min. The best-predicted oil yield of 7.29 g/100 g matched the actual oil yield (7.20 ± 0.13 g/100 g), being 84% of the oil yield obtained by Soxhlet extraction.

#### Kinetics of Ground Press Cake Maceration

Based on the observed extraction mechanism, the kinetics of oil maceration is described by the simplified phenomenological model (Stamenković et al., 2018):

(1) $$q=q_{\infty }\,\left[1-\left(1-f\right)\cdot e^{-k_{d}\cdot t}\right]$$

where *q* is oil yield at time *t*, *q*_*∞*_ is maximum oil yield at saturation, *f* is the fraction of oil extracted by washing (washable oil) and *k*_*d*_ is diffusion rate constant. This model supposes instantaneous oil washing, followed by oil diffusion. At *t* = 0, *f* = *q*/*q*_∞_.

The saturation oil yield, *q*_*∞*_, increases with an increasing extraction temperature and solvent:seed cake ratio due to increased oil solubility at higher temperatures and an increased amount of solvent that dissolves a larger amount of the oil, respectively. The washable oil fraction, *f*, increases with increasing solvent:seed ratio at a constant temperature due to the positive effect of the increased amount of solvent on washing. The diffusion rate constant, *k*_*d*_, increases with an increasing solvent:seed cake ratio and extraction temperature, which was attributed to the reduced viscosity of the liquid phase. Also, mass transfer was facilitated at higher solvent:seed cake ratios by the increased concentration driving force. The modified Arrhenius equation was used to correlate the diffusion rate constant with solvent:seed cake ratio and temperature. The activation energy value (5.99 kJ/mol) was close to that for the hempseed oil maceration by *n*-hexane (5.75 kJ/mol) (Kostić et al., 2014).

#### Thermodynamics of Ground Press Cake Maceration

Maceration thermodynamics involves analysis of enthalpy, entropy, and Gibbs free energy changes, as well as the temperature extraction coefficient. This analysis is based on oil content determined by the Soxhlet extraction, oil yield during press cake maceration, and oil content in the exhausted press cake at saturation, which are used to calculate the distribution coefficient (*K*_*D*_). According to the Van’t Hoff equation, the dependence of ln*K*_*D*_ on the reciprocal absolute temperature at different solvent:seed ratios is linear, allowing the calculation of enthalpy, entropy, and Gibbs free energy changes. The enthalpy and entropy changes for the maceration of ground press cake using *n-*hexane were positive, ranging from 5.2–12.5 kJ/mol and 29–47 J/(mol K), respectively (Stamenković et al., 2018). This implies that ground press cake maceration is endothermic and irreversible. Values of the enthalpy and entropy changes for oil maceration from ground white mustard cake are similar to those for oil extraction from olive cake, hemp seeds, and sunflower seeds but much lower than those for cotton seeds and soybean flakes (Table 4).

**TABLE 4:** Comparison of thermodynamic quantities for oil extraction from different oily materials.^a^

**Plant material**	***T* (°C)**	***ΔS°* (J/mol K)**	***ΔH°* (kJ/mol)**	***ΔG°* (kJ/mol)**	**References**
White mustard seed cake	20–70	29-47	5.2-12.5	−4.8 to −1.4	Stamenković et al., 2018
Olive cake	20–50	12.9	59.3	–6.3 to –4.5	Meziane and Kadi, 2008
Soybean flakes	50–100	48.2–95.4	137–296	–10 to –4	Rodrigues et al., 2010
Sunflower seeds	30–60	11.2	36.75–39.60	–1.1 to –0.8	Topallar and Gecgel, 2000
Cotton seeds	15–45	43.2–85.8	190.9–331.3	–21.0 to –10.4	Saxena et al., 2011
Hemp seeds	20–70	6.17–10.54	33.09–44.19	–5.17 to –2.41	Kostić et al., 2014

*^*a*^Adapted from Stamenković et al. (2018).*

The negative Gibbs free energy change (from –4.8 kJ/mol to –1.4 kJ/mol) means that the process is feasible and spontaneous (Stamenković et al., 2018). The spontaneity of ground press cake maceration is favored by an increasing solvent:seed cake ratios and maceration temperatures.

The temperature extraction coefficient defines the increase in oil yield for every 10°C increase in extraction temperature. For white mustard seed cake, its values were 1.040, 1.021, and 1.011 for maceration at solvent:seed cake ratios of 3:1, 6.5:1, and 10:1 mL/g, respectively (Stamenković et al., 2018). These values are similar to those reported for oil extraction from olive cake (1.02–1.14) (Meziane and Kadi, 2008) and hemp seeds (1.012–1.027) (Kostić et al., 2014).

### Oil Refinement

The final step in oil extraction is the refining process, which is comprised of several operations, including degumming, alkali treatment, bleaching, and deodorization (Vaisali et al., 2015). Degumming removes phosphatides and mucilaginous gum while the alkali-refining treatment eliminates free fatty acids, color bodies, and metallic pro-oxidants. Bleaching removes pigments and residual soaps and improves the taste of the oil. Deodorization is carried out through high-vacuum steam distillation to remove unwanted odor and taste from the degummed and/or neutralized oil.

### Fatty Acid Profile and Physicochemical Properties of White Mustard Oil

The oil contents, fatty acid profiles, and physicochemical properties of white mustard oil obtained from seeds and press cake by various extraction techniques are in Table 5. Generally, all oils contain the same fatty acids, thus indicating no influence of the extraction method on their composition. For seeds from Serbia, the content of total saturated fatty acids (SFA) is very low (2.0–4.1%) and increases by thermal treatment during the Soxhlet extraction (Stamenković et al., 2018). Among the SFAs, palmitic acid is the most abundant (about 0.7–3.4%), with an exceptionally high content of palmitic acid reported for a Canadian oil (23.7%). The monounsaturated fatty acids (MUFA) include oleic (C18:1), eicosenoic (C20:1), erucic (C22:1), and nervonic (C24:1) acids, with erucic acid the most abundant (32.8–60.3%). The high content of erucic acid is a unique property of white mustards originating from various regions, particularly Europe. Oleic acid is the second most abundant fatty acid with a content of 9.1–43.4%. The highest oleic acid content is characteristic for white mustard oils from North and South America. The primary polyunsaturated fatty acids (PUFA) of white mustard oils are linoleic (C18:2) and linolenic (C18:3) acids. The ratio of oleic to linoleic fatty acids (stability index) in white mustard oil depends on the extraction technique and decreases with thermal treatment as the content of linoleic acid increases. For the same reason, the linoleic acid/linoleic acid ratio increases after thermal treatment.

**TABLE 5:** Variability of oil content, fatty acid profile and physicochemical properties in white mustard seed and press cake of different origin.^a^

**Origin**	**Serbia**				**Romania**		**Spain**	**Great Britain**	**Israel**	**India**	**Canada**			**Mexico**
**Extraction**	**Soxhlet/**	**Cold**	**Soxhlet/**	**Maceration/**	**Soxhlet/**	**Cold**	**Soxhlet/**	**Soxhlet/**	**Soxhlet/**	**Soxhlet/**	**Soxhlet/**		**Cold**	**Cold**
**technique**	***n*–hexane**	**pressing**	***n*–hexane^b^**	***n*–hexane^b^**	***n*–hexane**	**pressing**	***n*–hexane**	**petroleum**	**petroleum**	***n*–hexane**	***n*–hexane**	**Commercial**	**pressing**	**pressing**
								**ether**	**ether**					
Yield, %	20.6	13.2	8.58	7.20	43.5	∼35	25	–	19.5	31.6	35.1	–	–	22.3
C14:0	–	–			–	–	–	–	–	–	0.3	0.05	–	–
C16:0	0.86	0.73	1.09	1.72	1.6	1.5	2.82	3.1	3.0	3.36	23.7	2.80	2.81	2.10
C16:1	–	–	0.11	0.14	–	–	–	–	–		–	0.16	–	0.09
C16:2	–	–			–	–	–	–	–		–	0.06	–	–
C18:0	0..35	0.3	0.38	0.61	0.7	0.9	–	0.7		1.12	1.6	1.09	1.06	0.80
C18:1	11.63	13.95	12.60	14.86	12.4	12.0	17.61	9.1	15.8	22.12	43.4	26.08	24.89	19.62
C18:2	6.03	5.98	7.46	8.86	12.0	12.3	7.82	11.7	9	10.78	30.1	11.64	9.21	8.43
C18:3	7.00	7.37	8.03	8.76	8.7	8.9	10.99	12.5	8.6	12.52	0.2	8.61	10.8	21.64
C20:0	0.33	0.28	0.35	0.52	0.7	0.6	–	0.7			0.6	0.70	–	0.41
C20:1	7.00	7.41	7.32	9.59	6.7	6.6	5	10.8	5.8	11.91	–	10.44	10.63	*nd*
C20:2	0.22	0.17	0.24	0.29	0.3	0.3	–	0.7	–		–	–	–	0.25
C22:0	0.57	0.39	0.56	0.78	0.7	0.6	–	Tr	–		–	0.57	–	–
C22:1	60.29	59.98	56.21	49.81	55.0	55.0	55.76	46.5	50.8	38.16	–	32.81	34.94	40.80
C22:2	0.40	0.32	0.42	1.26	0.5	0.4	–	0.4	–		–	–	–	–
C24:0	0.50	0.30	0.69	0.52	0.1	0.1	–	Tr	–		–	–	–	nd
C24:1	4.79	2.95	4.59	2.31	0.6	0.6	–	3.6	–		–	–	–	1.25
SFA, %	2.61	2.00	3.05	4.14	3.80	3.70	2.82	4.50	3.00	4.48	26.20	5.21	3.87	3.31
MUFA,	83.7	84.3	80.82	76.70	74.7	74.2	78.4	70.0	72.4	72.2	43.4	69.5	70.5	61.8
PUFA, %	13.7	13.8	16.14	19.17	21.5	21.9	18.8	25.3	17.6	23.3	30.3	20.3	20.0	30.3
ALC	20.9	20.8	19.5	20.8	20.4	20.4	20.3	20.2	18.8	19.7	17.5	18.6	18.5	18.8
TUD, %	118.0	119.3	121	119	126.4	126.9	127.0	133.1	116.2	131.3	104.2	118.4	121.3	144.0
OLR	1.93	2.33	1.69	2.13	1.03	0.98	2.25	0.78	1.76	2.05	1.44	2.24	2.70	2.33
LLR	0.86	0.81	0.93	0.84	1.38	1.38	0.71	0.94	1.05	0.86	–	1.35	0.85	0.39
AV	2.73	1.95	4.09	4.43	1.58	1.23	–	1.23	–	–	2.19-	0.85	–	–
SV	180.72	180.65	178.29	179.65	–	–	–	174	–	184.7	175	–	–	–
IV	101.78	100.58	107.49	108.21	102.3	102.3	–	105.4	–	112	106.2-	–	–	–
CV	–	–			–	–	–	–	–	50.6	–	–	–	–
Reference	Stamenković et al., 2018				Ciubota-Rosie et al., 2013		Sáez-Bastante et al., 2016	Ali and McKay, 1982	Yaniv et al., 1994	Singh et al., 2014	Sengupta and Bhattacharyya, 1996	Issariyakul et al., 2011	Nie et al., 2016	Mejia-Garibay et al., 2015

*^*a*^Adapted from Stamenković et al. (2018). SFA, saturated fatty acids; MUFA, monosaturated fatty acids; PUFA, polyunsaturated fatty acids; ALC, average length chain; TUD, total unsaturation degree; OLR, oleic/linoleic ratio; LLR, linoleic/linoleic (ω–6/ω–3) ratio; AV, acid value; SV, saponification value; IV, iodine value and CV; cetane value. ^*b*^Press cake.*

Generally, the extraction technique has little effect on the physicochemical properties of white mustard oil, except for the acid value, which is higher for oil obtained through solvent extraction (Stamenković et al., 2018). This is attributed to the higher temperature of solvent extraction affecting oil acidity caused by the hydrolysis of acylglycerols (Adeeko and Ajibola, 1990). However, the acid and iodine values of oils depend on the oily feedstock (seed or press cake), which are higher for the oil from press cake (Stamenković et al., 2018) due to the pressing, milling, and solvent extraction process that increases free fatty acid formation (Adeeko and Ajibola, 1990). The saponification value depends on neither extraction technique nor feedstock (Stamenković et al., 2018).

## Biodiesel Production From White Mustard Seed Oil

White mustard seed oil is a promising oily feedstock for biodiesel production (Ciubota-Rosie et al., 2013). In many countries, it is considered unsuitable for human consumption (Wendlinger et al., 2014). While white mustard seed can be used as a spice, its widespread use in the food industry is restricted by its strong, hot taste and high erucic acid content. Therefore, its use as an alternative feedstock for biodiesel production will not compete with its use as human food. Indeed, the transesterification of erucic acid provides alkyl esters with great lubricant properties for better engine operation (Issariyakul et al., 2011). Furthermore, white mustard can grow spontaneously on abandoned land or under cultivation, typically in rotation with cereal crops (Falasca and Ulberich, 2011; Rahman et al., 2018). It can also grow on different soil types, is resistant to many diseases and insect pests, and can endure extreme weather conditions without substantial harm (Sask Mustard, 2019). Considering the maximum grain production, the oil content and its conversion into methyl esters, Sáez-Bastante et al. (2016) estimated the white mustard oil-based biodiesel production of about 480-486 L ha^–1^. This biodiesel output is similar to the biodiesel production from soybean oil (400-500 L ha^–1^) but much lower than the outputs from sunflower (1,000 L ha^–1^), rapeseed (1,200 L ha^–1^), or palm (5,000 L ha^–1^) oil (Worldwatch, 2006). Recently, Jaime et al. (2018) have shown that white mustard could replace rapeseed for biodiesel production in the Mediterranean basin and other Western European countries where their cultivation is expected to be compromised by climate change. Another advantage of white mustard, compared to the other traditional feedstocks for biodiesel production, is the possibility îf its growth on abandoned land with marginal cultivation requirements.

The literature reveals that white mustard oil is used as a feedstock for biodiesel production through a transesterification reaction in the presence of homogeneous or heterogeneous base catalysts while no acid catalyst has been applied. The homogeneous base-catalyzed transesterification is suitable for biodiesel production from white mustard oil due to its low acid value. Indeed, biodiesel has been mainly produced from white mustard oil using homogeneous base catalysts, while a heterogeneous catalyst was applied only in a study (Table 6). It can be speculated that the researchers have preferred to use homogeneous base catalysts because they provide fast reactions and a high ester yield under mild reaction conditions, compared to both homogeneous acid and solid base catalysts. It may be expected that solid base catalysts will get more attention in the future because of their well-known advantages over homogeneous catalysts (for instance, easy recovery from the reaction mixture and reusability).

**TABLE 6:** Review of the alcoholysis reaction of white mustard oil.

						
**Type of alcohol**	**Alcohol:oil molar ratio**	**Catalyst/amount (% of oil)**	**Temperature (°C)**	**Type, volume of reactor/agitation speed (rpm)**	**Yield (purity) (%/time)**	**References**
Methanol	2:1–10:1	NaOH/0.2–1.0	50–70	Three-neck flask, 250 mL/magnetic/800 rpm	96.5%/ 62.12 min^a^	Yesilyurt et al., 2019
Methanol	6:1	KOH/1	40–60	Stirred reactor, –/600 rpm	–	Issariyakul and Dalai, 2012
Methanol	6:1	CH_3_OK/0.2:1^b^	60	Stirred reactor, 500 mL/–	(>98)/1.5 h; (99)/4 h	Ciubota-Rosie et al., 2013
Methanol	6:1	NaOH/0.8	70	Stirred reactor, 2 L/–	(82)/2 h	Ahmad et al., 2013
Methanol	2:1–10:1	NaOH/0.1–0.9	50–75	Stirred reactor, 500 mL/600 rpm	92/75 min^c^	Sultana et al., 2014
	6:1	KOH/0.5	65		84/75 min^c^	
Methanol^c^	14:1	NaOH/1.2	Room	Stirred reactor, 200 mL/magnetic	(99.3)/10 min^d^	Tabtabaei et al., 2015
Methanol	12% oil relative to KOH/methanol solution	KOH/0.5^e^	22	–	–	Nie et al., 2016
Methanol	25:6 mL/mL	KOH/1.8	65	Erlenmeyer flask/magnetic/300 rpm	96.56/2 h	Oshodi et al., 2014
Methanol	6:1	KOH/0.3	60	–	–/2 h	Sarala et al., 2012
Methanol Ethanol, propanol, 1-butanol	6:1	KOH/1 CH_3_ONa/0.5 and 1	60	Stirred reactor, –/600 rpm	(66)/1.5 h^f^	Issariyakul et al., 2011
					(66)/1.5 h^f,g^	
Methanol	–	NaOH (150 mL, 1 M)	55	Glass container, jerked/–	–/5 min	Alam and Rahman, 2013
Methanol	6:1–8:1	NaOH/0.7	60	–	85%/8:1	Ahmad et al., 2008
Methanol	6:1	KOH/1	60	Three-neck flask, 250 mL/magnetic/400 and 900 rpm	(98.7%)/20 min	Kostić et al., 2018
	6:1–12:1	Quicklime/2–10			(98.5%)/50 min^h^	

*^*a*^Optimal conditions (methanol:oil molar ratio 7.41:1, NaOH 0.63% of oil, 61.84°C); ^*b*^Catalyst:oil molar ratio; ^*c*^Optimal conditions (methanol:oil molar ratio 6:1, NaOH 0.5% of oil, 65°C); ^*d*^In the presence of THF (THF:methanol 1:1 mL/mL); ^*e*^wt% to methanol mass; ^*f*^Methanol, two-step process with glycerol removing between steps; ^*g*^1% CH_3_ONa; ^*h*^Optimal conditions (methanol:oil molar ratio 12:1, CaO 10% of oil) and after ester purification*

A summary of biodiesel production from white mustard oil is in Table 6. In these reactions, triacylglycerols (TAG) from white mustard oil are converted into fatty acid alkyl esters, most frequently fatty acid methyl esters (FAME). Generally, as is the case for other oily feedstocks (Živković et al., 2017), the reaction efficiency and ester yield are influenced by many factors, including the type of alcohol, initial alcohol:oil molar ratio, type and amount of catalyst, reaction temperature, mixing intensity, and reaction time.

### Base-Catalyzed Transesterification of White Mustard Seed Oil

To date, biodiesel production from white mustard oil has mainly used homogeneous base catalysts (Table 6). Alkali hydroxides (KOH and NaOH) are more frequently used than alkali methoxides. NaOH is catalytically more active than KOH, providing higher ester yields (92% vs. 84%) under the same reaction conditions (Sultana et al., 2014). The use of catalysts, ranging from 0.1 to 1.8% (based on oil mass), under different reaction conditions resulted in various methyl ester yields, making it difficult to select the optimal catalyst concentration. Generally, low catalyst amounts will not complete the reaction, while excess catalyst amounts favor soap formation, both of which result in lower ester yields (Yesilyurt et al., 2019). Catalyst amounts of about 1% (based on oil mass) are used most often. Methanol is the main alcohol used, but others include ethanol, propanol, and 1-butanol (Issariyakul et al., 2011). The methanol:oil molar ratio ranges from 2:1 to 14:1, with 6:1 the most frequently applied ratio. At lower methanol amounts, the reaction reaches equilibrium at lower FAME contents, whereas higher amounts result in faster reactions and higher final ester contents. This is attributed to a shift in the reaction equilibrium toward TAG conversion and the reduced density and viscosity of the reaction mixture (Kostić et al., 2018). However, excess alcohol can cause difficulties in glycerol separation from the esters phase, lowering FAME yield (Yesilyurt et al., 2019). Glycerol formation has a small effect on ester yield, as the two-step reaction with glycerol removal in between only increased ester yield by 2% (Issariyakul et al., 2011). The transesterification reaction is carried out at different temperatures (22–75°C) but most frequently at close to alcohol boiling point. The FAME content in the esters phase is dependent on the quality of the white mustard oil (Ciubota-Rosie et al., 2013) and the reaction conditions. Generally, FAME purity and yields are higher with commercial (refined) oils (Oshodi et al., 2014) than crude oils (Ahmad et al., 2008, 2013; Sultana et al., 2014). Ciubota-Rosie et al. (2013) reported that achieving FAME contents above 80% was difficult with crude oil due to the presence of phosphorus compounds, gums, and free fatty acids that emulsify or cause sediment, making ester synthesis, separation, and purification difficult.

Quicklime is the only heterogeneous catalyst that has been used for methanolysis of white mustard oil (Kostić et al., 2018). At optimal reaction conditions, the quicklime-catalyzed reaction is slower than the KOH-catalyzed reaction due to greater mass-transfer limitations in the three-phase reaction system, which controls the overall process rate. A heterogeneous reaction carried out with a methanol:oil molar ratio of 12:1 and 10% quicklime (based on oil mass) for 50 min provided almost the same TAG conversion as the KOH-catalyzed reaction under milder reaction conditions for a shorter time (methanol:oil molar ratio 6:1, 1% KOH, and 20 min). However, heterogeneous reactions are a cheaper, more straightforward, and more environmentally friendly process (Kostić et al., 2018).

### Modeling and Optimization of Biodiesel Production From White Mustard Oil

Useful tools for improving biodiesel production from any oily feedstock include statistical modeling and optimization. Ester yield is influenced by the reaction conditions, namely initial alcohol:oil molar ratio, catalyst type and loading, reaction temperature, and time. Therefore, it is important to know the impact of these process factors on ester yield and optimization. Response surface methodology (RSM), combined with specific experimental designs, has been widely used to optimize biodiesel production from various oily feedstocks, but rarely for improving white mustard oil-based biodiesel production (Kostić et al., 2018; Yesilyurt et al., 2019).

Yesilyurt et al. (2019) applied a central composite design combined with the RSM to analyze and optimize methanolysis of white mustard oil catalyzed by NaOH in an experimental domain (methanol:oil molar ratio 2:1–10:1, NaOH loading 0.2–1.0% of oil mass, reaction temperature 50–70°C, and reaction time 30–90 min). According to the developed second-order polynomial model, the methanol:oil molar ratio, temperature, and time had a significant, positive influence on ester yield, while NaOH loading was statistically insignificant. All the quadratic terms and the interaction of methanol:oil molar ratio with reaction time had significant, adverse effects on ester yield, while the interactions of catalyst loading with reaction temperature and reaction time were significant and positive. The optimal reaction conditions for the highest ester yield were methanol:oil molar ratio 7.4:1, catalyst concentration 0.63 wt.%, reaction temperature 61.84°C, and reaction time 62.62 min. The predicted FAME yield of 96.7% agreed with the experimental value of 96.5%. The quicklime-catalyzed methanolysis of white mustard oil was statistically analyzed and optimized for the methanol:oil molar ratio (6:1–12:1), catalyst amount (2–10%, of oil mass), and reaction time (30–50 min) using to 3^3^ full factorial design with replication combined with RSM (Kostić et al., 2018). The experimental data were modeled by a quadratic model, the adequacy and reliability of which was proven by statistical criteria. All three individual factors, as well as the interactions of catalyst amount with methanol:oil molar ratio and reaction time and the quadratic terms for catalyst amount, had a significant influence on ester yield. All three factors also had a positive effect on the FAME content. Based on the reduced quadratic model, complete TAG conversion could be obtained at catalyst amounts >9.8% and methanol:oil molar ratios ranging from 6.1:1 to 11.6:1 for 50 min.

### Kinetic Modeling of Biodiesel Production From White Mustard Seed Oil

Although reaction kinetics is fundamental for process design and development, the kinetics of white mustard oil transesterification has been rarely studied (Issariyakul and Dalai, 2012; Kostić et al., 2018). To develop a kinetic model for white mustard oil methanolysis catalyzed by KOH, Issariyakul and Dalai (2012) proposed a mechanism involving three consecutive, reversible reactions following the second-order reaction rate law. To avoid mass transfer limitations in the initial reaction period, the reaction mixture was vigorously stirred (600 rpm). The reaction rate constants of the forward reactions were at least one order of magnitude higher than the reverse rate constants. Increasing the temperature increased the forward reaction constants, while the effect of temperature on the reverse reaction rate constant was more complex. Based on the values of the rate constants, it was concluded that the rate-determining step was the conversion of TAG to diacylglycerides with an activation energy of 26.8 kJ/mol. The lower activation energy of the white mustard oil methanolysis reaction, compared to palm oil methanolysis (30.2 kJ/mol), was attributed to its lower content of saturated fatty acids. Agreement between experimental and predicted data confirmed the reliability and accuracy of the developed kinetic model.

Kostić et al. (2018) used two different reaction mechanisms for white mustard oil methanolysis over quicklime: (a) the first-order reaction with respect to TAG in the heterogeneous and pseudo-homogeneous regimes and (b) the changing mechanism combined with the TAG mass transfer limitation. The first model confirmed that TAG mass-transfer controlled the reaction in the initial, heterogeneous reaction regime, while in the later pseudo-homogeneous regime, the chemical reaction is the rate-determining step. The volumetric TAG mass-transfer coefficient was dependent on the initial catalyst and methanol concentrations, while the apparent reaction rate was constant. The second model, which combined the changing mechanism and first-order reaction rate law with respect to TAG and FAME, was also verified for the whole reaction period. Its parameters—reaction rate constant and “pure” TAG affinity for the active catalyst sites—were lower than those determined for sunflower oil methanolysis over quicklime, which was attributed to differences in the composition of the oily feedstocks. The mean relative percentage deviations of the TAG conversion degree for the more straightforward and more complex models were 3.0% and 16.1%, respectively, indicating the validity of both kinetic models. Therefore, while the more straightforward model is not applicable in the middle reaction period, it can be successfully used for simulation of white mustard oil methanolysis over quicklime.

## Fuel Properties of White Mustard-Based Biodiesels

The most important physicochemical and fuel properties of white mustard-based biodiesels reported in the literature, and standard biodiesel properties according to EN14214, are in Table 7. Generally, most of the properties fulfill the standard biodiesel quality, except for purified biodiesels obtained from NaOH- and CH_3_OK-catalyzed methanolysis of white mustard oil. The flashpoint, sulfur content, and acid value of white mustard-based biodiesels obtained using NaOH were not in accordance with EN14214 but satisfied the ASTM standard (Ahmad et al., 2013; Sultana et al., 2014). Since there are no reports on exhaust gas emissions for white mustard oil-based biodiesel, this important issue is not considered here.

**TABLE 7:** Properties of purified biodiesel obtained by base-catalyzed transesterification of white mustard oils.

	**Catalyst (conc.)/Alcohol**									
**Property**	**CaO (10%^a^)**	**KOH (1%^ a^)**	**KOH (1% ^a^)**				**NaOH (0.5%^ a^)**	**NaOH (0.72%^ a^)**	**CH_3_OK (0.2:1^b^)**	**EN14214 limit(min/max)**
	**Methanol**	**Methanol**	**Methanol**	**Ethanol**	**Propanol**	**Butanol**	**Methanol**	**Methanol**	**Methanol**	
FAME content (%)	98.9	98.7	99.8	99.7	99.7	98.0		82	<98	96.5 min
Density at 15°C (kg/m^3^)	881.1	880.1	900	900	900	900	834.3	899	878	860/900
Viscosity at 40°C (mm^2^/s)	4.15	4.13	4.2	4.5	5.0	5.5	5.45	6.72	5.67	3.50/5.00
Flash point (°C)							90	110	178	101 min
Sulfur content (mg/kg)			0	14.7	9.5	10.4	43.2	130	0.21	10 max
Cetane number									60	51 min
Water content (mg/kg)	235	217	231	62	187	345			223	500 max
Oxidation stability at 110°C (h)									2	6.0 min
Acid value (mg KOH/g)	0.44	0.47	0.4	0.5	0.6	4.0	0.8		0	0.50 max
Iodine value (g I_2_/100 g)	102.9	104.7							102.3	120 max
Methanol content (%)									0	0.20 max
Monoglyceride content (%)	0.5	0.4							0.15	0.80 max
Diglyceride content (%)	0.1	0.1							0.05	0.20 max
Triglyceride content (%)	0.2	0.2	0	0	0	0			0.02	0.20 max
Free glycerol (%)									0.0008	0.02 max
Total glycerol (%)			0.121						0.05	0.25 max
Group I metals (Na+K) (mg/kg)		3.7								5.0 max
Group II metals (Ca+Mg) (mg/kg)	15.5									5.0 max
Phosphorus content (mg/kg)			9	4	10	8			0.87	4.0 max
Cold filter plugging point (°C)									-5	-5 max
Cloud point (°C)							-10	3	5	Not specified
Pour point (°C)							-13	-6		Not specified
Reference	Kostić et al., 2018		Issariyakul et al., 2011				Sultana et al., 2014	Ahmad et al., 2013	Ciubota-Rosie et al., 2013	

*^*a*^Catalyst:oil weight ratio, ^*b*^Catalyst:oil molar ratio.*

The higher biodiesel viscosity than the EN14214 standard limit, which could damage the injection system due to poorer atomization of the fuel spray, was attributed to the high molecular weight and large chemical structure of pure biodiesel B100 (Ahmad et al., 2013; Sultana et al., 2014) and the presence of long FAMEs (mainly methyl erucate), which comprised more than 60% of the mustard oil fatty acid profile (Ciubota-Rosie et al., 2013). Due to the lower oxidation stability (2 h) of white mustard oil-based biodiesel than the minimum required by the EN standard (6 h), antioxidants should be added (Ciubota-Rosie et al., 2013). The amount of group II metals (Ca + Mg) in the purified biodiesel from the quicklime-catalyzed white mustard oil transesterification was above the EN 14214 standard limit (Kostić et al., 2018). Hence, the biodiesel purification process should be improved to reduce calcium and magnesium contents further.

Sáez-Bastante et al. (2016) predicted important biodiesel properties using mathematical models based on the chemical properties of hydrocarbon chains. The prediction values for cetane number, density, and cold filter plugging point agreed well with the European standard limits, but kinematic viscosity did not. An increase in the unsaturation degree (i.e., concentrations of linoleic and linolenic acid) improves some biodiesel properties, such as kinematic viscosity and cold filter plugging point, and reduces others, such as cetane number, calorific value, and oxidation stability (Sáez-Bastante et al., 2016). Therefore, a compromise is needed to use white mustard oil as a feasible feedstock for biodiesel production.

The use of different esters (methyl, ethyl, propyl, and butyl) derived from white mustard oil by homogeneous base-catalyzed transesterification was tested for diesel additives (Issariyakul et al., 2011). Most of the properties of the distilled methyl, ethyl, and propyl esters satisfied the European (EN14214) and United States (ASTM D 6751) specifications, but butyl esters had higher acid values than the proposed limits. All of these esters showed potential as a lubricant additive for diesel fuel, particularly methyl esters. Moreover, the diesel/biodiesel blend had a higher lubricant potential than commercial diesel.

## Other Products and Uses of White Mustard

The white mustard plant—aerial parts, seeds, oil and oil components, and essential oil—has a variety of applications in agriculture, food, and other industries, including medicine, culinary, and phytoremediation. The most important uses for white mustard are intercropping (Farooq et al., 2011; Paulsen, 2011; Rahman et al., 2018), biofumigation (Viuda-Martos et al., 2007; Arriaga-Madrid et al., 2017; Berlanas et al., 2018), phytoremediation (Kos et al., 2003; Jankowski et al., 2014; Popoviciu et al., 2017; Bulak et al., 2018), oilseed crop with high-quality properties (Raney et al., 1995), as a protein and amino acid source (Bell et al., 2000; Sarker et al., 2015), and as a condiment crop (Katepa-Mupondwa et al., 2005). White mustard oil also has many industrial applications, such as the production of bio-polyols for synthesis of rigid polyurethane-polyisocyanurate foams (Paciorek-Sadowska et al., 2018), edible biopolymer films for food packaging (Hendrix et al., 2012), and particle and interior boards, including furniture (Dukarska et al., 2011). Also, non-edible white mustard seed oil is used as a lubricant and for lighting (Falasca and Ulberich, 2011). The essential oil isolated from white mustard seeds has the potential for food preservation (Graumann et al., 2008). Being a potent natural antioxidant, white mustard seeds can be used to treat many diseases involving free radicals (Thangi et al., 2016). White mustard has many medicinal uses, such as an emetic and diuretic, as well as for treating inflammatory conditions (arthritis and rheumatism), cardiovascular disease, cancer, and diabetes (Khan and Abourashed, 2010); however, there are limited clinical trials to support its use for any indication (Anonymous, 2019). Some parts of the plant can be used as forage, lignocellulosic raw material (Dukarska et al., 2011), green manure (Krstić et al., 2010), or biomass fuel (Maj et al., 2019). Maj et al. (2019) assessed the biomass energy traits of various crop species, including white mustard, intended as forecrops with low gross and net calorific values (lower than agro-biomass or forest biomass). White mustard had the highest heat of combustion (15.55 MJ/kg), indicating its potential as an additional bioenergy source.

## Economic, Environmental, and Social Considerations of White Mustard Cultivation, Processing, and Utilization

Besides technical issues, economic, environmental, social, human health risk/toxicological, and policy aspects are the main issues when assessing the cost-effectiveness and sustainability of white mustard cultivation, processing, and utilization. A few studies have investigated the socio-economic implications of white mustard cultivation and processing (Withers et al., 2000; Sharma, 2015; Vach et al., 2016), but only Tabtabaei et al. (2015) has reported on white mustard oil-based biodiesel production costs.

According to Withers et al. (2000), white mustard is a viable alternative crop for crop rotations for at least three reasons: (a) better utilization of existing equipment on farms, (b) increased diversification, and (c) contribution to weed and disease control with fewer chemicals than other crops. Sharma (2015) analyzed the socio-economic characteristics of mustard growers, cost and return of both mustard cultivation and oil production, and profitability of different patterns of marketing, and suggested how to increase the economic viability of mustard cultivation in the Morena district, India. Mustard cultivation and processing is profitable on every scale, with opportunities to increase yield and profit through the efficient management and adoption of improved seed varieties, fertilizers, and plant-protective chemicals. Vach et al. (2016) investigated the economic efficiency of three crops (white mustard, winter wheat, and spring barley) cultivated using conventional, conservation with minimum tillage, and no-tillage methods. No-tillage produced the highest average white mustard seed yield but seed yields in other tillage methods were not significantly lower. Among the tested crops, white mustard had the lowest profitability. Regarding tillage methods, cost-effective and easy-to-manage systems with lower tillage intensity level should be prioritized.

A major environmental benefit of oil recovery from white mustard seeds is related to the reduction in waste generation. An oil press unit produces no pollutants and thus has no direct relation to environmental benefits. However, any improvements in the efficiency of energy uptake by these units will have indirect environmental benefits, such as reduced electricity consumption. The only waste from the oil press unit is the solid press cake (meal), which can be used as an animal feed, for compost, or as a solid biofuel.

Tabtabaei et al. (2015) analyzed the preliminary costs of an integrated process for producing food-grade protein products, high-purity methyl esters, and fiber-rich solid residue from dehulled white mustard seed flour. This process involved two-stage aqueous processing of white mustard flour at pH 11 followed by membrane separation technologies to produce protein products, namely soluble and precipitated protein isolates, from the protein-rich skim fraction (Tabtabaei and Diosady, 2012, 2013). This analysis compared the cost of white mustard seeds with the values of the protein isolates as primary products and the biodiesel and fiber-rich residue as byproducts (Tabtabaei et al., 2015). While the ultimate process will be selected on its total implementation costs, the minimum requirement for process viability is that the cost of the products recovers the cost of the raw materials.

In the absence of studies directly related to white mustard seed oil-based biodiesel production, many reports on the production and use of biodiesel from other feedstocks can be used to estimate socio-economic, environmental, and toxicological implications (Živković et al., 2017). As biodiesel from other oily feedstocks, white mustard oil-based biodiesel is expected to have several positive impacts on sustainable development, including improvements in energy security, stimulation of economic development, and contribution to environmental protection. The basic requirements for success regarding these impacts include defining policy, objectives, tasks, operating manuals, responsible workers, and deadlines for each step in the manufacturing process.

Economic, environmental, and social implications of white mustard seed oil-based biodiesel production are expected to be the same or similar to those of other oilseed crops (Živković et al., 2017). First, white mustard oil, as a renewable source, will contribute to the substitution of non-renewable diesel fuel. White mustard plants can mitigate climate change by consuming CO_2_—a dominant greenhouse gas—during photosynthesis, reducing the negative impacts on air, water, land, and biodiversity, and promoting rural economic development. Second, the use of non-edible white mustard oil as a feedstock for biodiesel production does not contribute to the food versus fuel controversy. Finally, biodiesel production from white mustard oil will be closely connected to agricultural production and may contribute to energy security.

Characteristic pollution parameters during the production of white mustard oil should be similar to those of other crops (Živković et al., 2017). The increased use of mineral fertilizers causes ecological damage, reduces the quality of water for human consumption, and pollutes waterways. In addition, the energy embedded in chemicals (fertilizers, agrochemicals, and methanol) must be included in the life cycle assessment of white mustard-based biodiesel. Non-renewable energy—consumed during white mustard cultivation and processing, oil extraction, biodiesel production and purification, transportation of raw materials, inputs and distribution—must be considered when evaluating the overall influence of biodiesel on the environment. There are no published life cycle analyses of white mustard oil-based biodiesel production and use.

## Potential and Future Outlook of White Mustard Oil for Biodiesel

White mustard plant parts, especially seeds, have potential for economically valuable applications and research on their practical application as a bioenergy and bioproduct source. Innovative research will help to improve biodiesel production from white mustard oil and contribute to developing novel processes to produce other biofuels and value-added products. Further investigations are needed to reflect the kinetics of the transesterification of white mustard oil and optimize this reaction for the type and concentration of alcohol and catalyst, type of reactor, and reaction temperature. It is especially important to test the low-cost, active, and stable solid catalysts obtained from natural or waste sources using continuous operation. Novel biodiesel production processes, including unconventional heating methods (ultrasonication and microwave irradiation), continuous reactors with improved mass transfer characteristics, and integration of reaction and separation phases in a single stage, should be developed to advance the economy of the overall biodiesel production process. Possible uses of other parts of the white mustard plant to produce other types of biofuels by liquefaction, gasification, and pyrolysis should be evaluated. These methods face significant challenges for the commercial utilization of white mustard biomass for biofuel production. The generation of high-value products from white mustard should be considered. New emerging technologies that synergistically combine various conversion processes and provide multiple products, called biorefineries, are expected to address the technical and economic obstacles of existing biomass conversion processes. Biorefineries need to develop or improve constituent processes to optimize the integrated system, and provide heat and power supplies for, at least, energy self-sufficiency.

Research is needed to optimize suitable biomass properties without compromising the ability of white mustard plants to grow in diverse environments. Natural genetic variation could be used to improve the bioenergy properties of white mustard plants. In addition, agricultural management (including fertilizer type, time of harvesting, and biomass storage) is critical, and will impact biomass properties. Finally, intensive cultivation of white mustard is needed for making it attractive and economically favorable for biodiesel production on a commercial basis. This is especially important for the Mediterranean basin and Central Europe, as they are expected to become unsuitable for rapeseed in the near future, as shown by the models developed by Jaime et al. (2018). According to these models, the increases in aridity and average annual temperature will expand the climatically appropriate areas for the white mustard cultivation in the Mediterranean basin while favorable areas for the rapeseed cultivation will reduce remarkably in Western Europe. Because of its good potential as a biofuel crop and with potential for genetic improvements, white mustard could replace rapeseed crops for future biodiesel production in the above areas.

## Conclusion

This review considers all stages of biodiesel production from white mustard seed oil, from seed harvest, drying, storage, and pretreatment via oil recovery to transesterification reaction. White mustard seed oil is a promising feedstock for biodiesel production for several reasons: (a) plants can be cultivated on different soil types, usually in rotation with cereal crops, resists many diseases and insect pests, and endures extreme weather conditions without substantial harm, (b) the oil is considered unsuitable for human consumption in many countries due to its high erucic acid content, (c) the biodiesel has excellent lubricant properties for better engine operation, and (d) biodiesel production can be integrated with protein and oil recoveries into an economically justified process.

## Author Contributions

PM, OS, IB-I, ID, ZN, MF, KS, and VV wrote the manuscript.

## Conflict of Interest

The authors declare that the research was conducted in the absence of any commercial or financial relationships that could be construed as a potential conflict of interest.
